# Comparative accuracy analysis of robotic and static guided implant surgery: a retrospective clinical study

**DOI:** 10.1186/s12903-025-07625-4

**Published:** 2026-01-09

**Authors:** Fei Zhong, Gang Chen, Binbin Sun, Fengping Wu, Yulin An

**Affiliations:** 1 Multidisciplinary Consultation Center, Zhen Jiang Stomatological Hospital, Zhenjiang, 212002 China; 2 Department of Implant and Prosthetic Dentistry，Zhen Jiang Stomatological Hospital, Zhenjiang, 212002 China; 3 Science and Technology Innovation Center，Zhen Jiang Stomatological Hospital, Zhenjiang, 212002 China

**Keywords:** Robotic implantation, Surgical guide, Oral implantology

## Abstract

**Objective:**

This study aimed to evaluate the accuracy of robot-assisted implantation by comparing it with guide-assisted implantation in different surgical contexts, including immediate implantation, multi-tooth implantation, maxillary sinus lift, and bone defect.

**Methods:**

Patients receiving implantation with static guide-assisted implant procedures or robot-assisted procedures were classified into the guide group (30 patients, 31 implants) and robot group (30 patients, 32 implants), respectively. Pre- and post-operative CBCT scans were processed with software for 3D reconstruction. Deviations between the original plan and actual implant placement at the apical level, cervical level, and implant angulation were projected into mesio-distal and bucco-lingual directions respectively, and were measured for analysis along with depth discrepancy.

**Results:**

Comparing with guide group in this study, the robot group enjoyed smaller angular deviations in both bucco-lingual direction (1.289° ± 1.067 vs. 0.483° ± 0.334, P<0.01) and mesio-distal direction (1.652° ± 1.421 vs. 0.509° ± 0.426, P<0.01). Deviation analysis also demonstrated the advantage of robot system in controlling deviation at cervical (B-L: 0.461 ± 0.310mm vs. 0.183 ± 0.190mm, P<0.01; M-D: 0.441 ± 0.231mm vs. 0.121 ± 0.125mm, P<0.01) and apical distance (B-L: 0.508 ± 0.330mm vs. 0.164 ± 0.154mm, P<0.01; M-D: 0.476 ± 0.346mm vs. 0.135 ± 0.124mm, P<0.01). No important difference was noted in depth accuracy (0.426 ± 0.205, 0.335 ± 0.029, P>0.05). Notably, the precision of robotic implant placement remained consistently high across various procedures, including conventional, immediate, bone defect, class IV bone, sinus elevation, and multiple-tooth placements, with no important variation.

**Conclusion:**

Compared to guide-assisted techniques, the robotic implantation system offers superior accuracy in clinical implanting contexts. Its integration allows surgeons to focus more on planning and evaluation workflow, reducing manual effort and potentially lowering labor costs.

**Trial registration:**

Full name of the registry: Chinese Clinical Trial Registry. Trial registration number: ChiCTR2500109094. This study was retrospectively registered at Chinese Clinical Trial Registry (www.chictr.org.cn) on 11^th^ Sept 2025.

## Introduction

Since the advent of dental implantology, there has been a continuous pursuit of optimizing the implantation procedure and achieving precise prosthetic positioning. The evolution of implant surgery has progressed through distinct phases: freehand surgery, image-guided surgery, static guide-assisted surgery (also known as static navigation), and navigation-assisted surgery [1]. Robotic-assisted implant surgery represents the latest phase in the evolution of dynamic navigation [2].

In the pre-digital era, implant placement relied solely on the surgeon’s freehand technique, devoid of computer-aided planning. This approach was associated with significant risks and poor predictability, often leading to complications [3, 4]. The inherent limitations of freehand surgery, including challenging intraoperative decision-making and low reproducibility, catalyzed the development of digital navigation technologies.

In early 1990s, the introduction of three-dimensional dental software popularized the use of software and tomographic images for guiding implant surgery [5]. This evolution led to the development of image-guided techniques utilizing surgical templates to direct osteotomy preparation with precise depth and orientation. A significant milestone in this technological evolution was the emergence of static guide-assisted implant surgery. The earliest static guides were fabricated by casting and milling techniques, where the implant trajectory was machined into solid casts [6]. With advancements in additive manufacturing and materials science in the new century, 3D-printed static guides have gradually become the mainstream technology [7].

The principle of static navigation involves the fusion of digital scan data with a patient-specific oral model, enabling precise virtual planning of implant position and trajectory [8]. Surgical guides, fabricated via 3D printing based on preoperative CBCT data, encode the planned spatial parameters. While static guides have markedly improved accuracy over freehand placement and become a cornerstone of contemporary implantology [9], they possess inherent drawbacks: dependency on the accuracy of CBCT and intraoral scan data fusion, risk of guide fracture or deformation, inability to intraoperatively deviate from the preplanned path, and susceptibility to errors introduced by guide sleeve tolerance and limited patient mouth opening [8, 10].

The introduction of dynamic navigation, particularly computer-assisted surgery utilizing optical tracking, mitigated some limitations of static guidance [11]. This passive navigation technology provides surgeons with real-time, dynamic visualization of surgical instrument and implant positions on a monitor, allowing for intraoperative verification and limited trajectory adjustment [12]. It achieves accuracy comparable to or exceeding static guidance [10]. However, as a passive system, it still necessitates manual execution by the surgeon and does not eliminate errors arising from hand tremor or manual dexterity [13].

Robot-assisted surgery emerged as a transformative active navigation technology. The first FDA-approved dental robot system was applied in 2017, and in the same year the first Chinese autonomous robotic platforms was also launched, which were used in this study [14]. It integrates a surgical navigation platform, a commercial robotic manipulator, a dedicated implant platform, and the DentalNavi planning software. Following a surgeon-supervised registration and potential path “learning” phase, the robotic arm can autonomously execute key surgical steps like drilling and implant insertion. A key advantage of robotic navigation is its liberation of the surgeon’s hands from manual execution, thereby allowing a focus on procedural planning, assessment, and guiding the robotic arm’s path, while also mitigating several inherent limitations of static guide-assisted surgery:

1. Elimination of Physical Guide Constraints: It obviates the requirement for a physical guide, thereby removing issues related to guide fit, stability, fracture, and restricted access and can execute complex, pre-programmed movements that may exceed human physical capabilities.
2. Enhanced Execution Fidelity: As an active system, the robotic arm executes the preoperative plan with high mechanical precision, effectively eliminating the influence of human hand tremor and manual execution errors inherent in freehand and passive dynamic navigation.
3. Retention of Intraoperative Adaptability: Unlike static guides, which lock the surgeon into a single plan, robotic systems combine high precision with the potential for real-time tracking and intraoperative plan modification, preserving surgical flexibility.
4. Precise calculations facilitate rapid optimization: Unlike static guides, the robotic system operates based on pre-programmed digital protocols. Processes such as registration, drilling, feedback, and correction are all governed by precise computational algorithms. Any procedural inaccuracy or deviation can be precisely and timely identified, enabling surgeons to promptly make corrections, thereby enhancing implantation accuracy.

Studies on several robotic systems application have revealed their feasibility in various implantation scenarios [15–18]. However, there is a notable paucity of robust clinical studies directly comparing the accuracy of robot-assisted surgery against the static guide-assisted approach, particularly in live clinical scenarios.

In brief, the evolution from freehand to static, dynamic, and robot-assisted surgery marks a clear trajectory toward enhanced precision, control, and predictability in implant dentistry. Robotic systems represent a paradigm shift by actively executing the surgical plan, thereby overcoming the physical constraints of static guides. The goal of this study is to compare how accurately robotic implantation system performs against static guide in real clinical contexts—including traditional and immediate implant placements, procedures on bones with deficiencies, cases involving type IV bone, maxillary sinus floor augmentations, and multi-tooth implantations in a row. We aim not only to evaluate the precision of these techniques, but also to improve the workflows involved in robotic surgeries, understand their technical boundaries in real clinic settings, and finally enhance their value in clinical practice.

## Materials and methods

### Case selection

In this observational study, all data were drawn from patients undergoing existing treatments in Zhenjiang Stamotological hospital. A total of 30 guide-supported implant cases performed from November 2024 to March 2025 were assigned to the guide group. Also, 30 cases of robotic implant surgeries using the Yazhi Dental Implant Robotic System (Yake Wisdom System, Beijing, China), conducted between September 2023 and December 2023, were categorized as the robot group (Table 1). All cases involved the Osstem TS Ⅲ SA implants (Osstem Implant Co., Ltd., South Korea), with 31 and 32 implants for guide and robot group respectively (Table 2). Minimally invasive, flapless procedures were employed for all surgeries unless guided bone regeneration (GBR) was necessary, in which case flap procedures were performed. According to the Misch classification, bone density values analyzed by SIDEXIS software (Sirona Dental Systems GmbH, Germany) were categorized as follows: densities below 350 HU were classified as Type IV, and those above 1250 HU were classified as Type I.

**Table 1:** Demographic characteristics of both groups

	Gender		Age (year-old)			
	Male	Female	<41	41 ~ 50	51 ~ 60	>61
Robot Group(*n* = 30)	16	14	2	3	4	20
Guide Group(*n* = 30)	20	10	2	1	8	18

**Table 2:** Implant distribution in both groups

	Maxilla	Mandible	Anterior Region	Posterior Region	Type I bone	Type II-III bone	Type IV bone
Robot Group(*n* = 32)	18	14	12	20	3	24	5
Guide Group(*n* = 31)	16	15	13	18	1	27	3

Healing abutments were placed following implant position stabilization. All operations were conducted by a dedicated surgical team comprising an experienced implantologist (the first author, 12 years of experience) and a surgical nurse. This project was approved by the Ethics Committee of Zhenjiang Stomatological Hospital (PJ2023-09-01).

#### Inclusion criteria

1. Bone height of at least 4 mm and width of at least 5 mm;
2. Bone quality classified as types I through IV;
3. Mouth opening of 35 mm or more, suitable for robotic surgery; absence of temporomandibular joint dislocation history;
4.  Guide supportability based on remaining teeth for the guide group;
5. No contraindicating health conditions such as infectious or hematological diseases.

#### Exclusion criteria

1. Systemic conditions (chronic or genetic) contraindicated for implant surgery;
2. Surgical procedures that entailed the placement of zygomatic, pterygoid, or orthodontic mini-implants;
3. Heavy smoking or active periodontitis.
4. Compromised stability of the static surgical guide due to mobility of the remaining teeth or tooth defects.

### Components of the robotic system

**Fig. 1:**
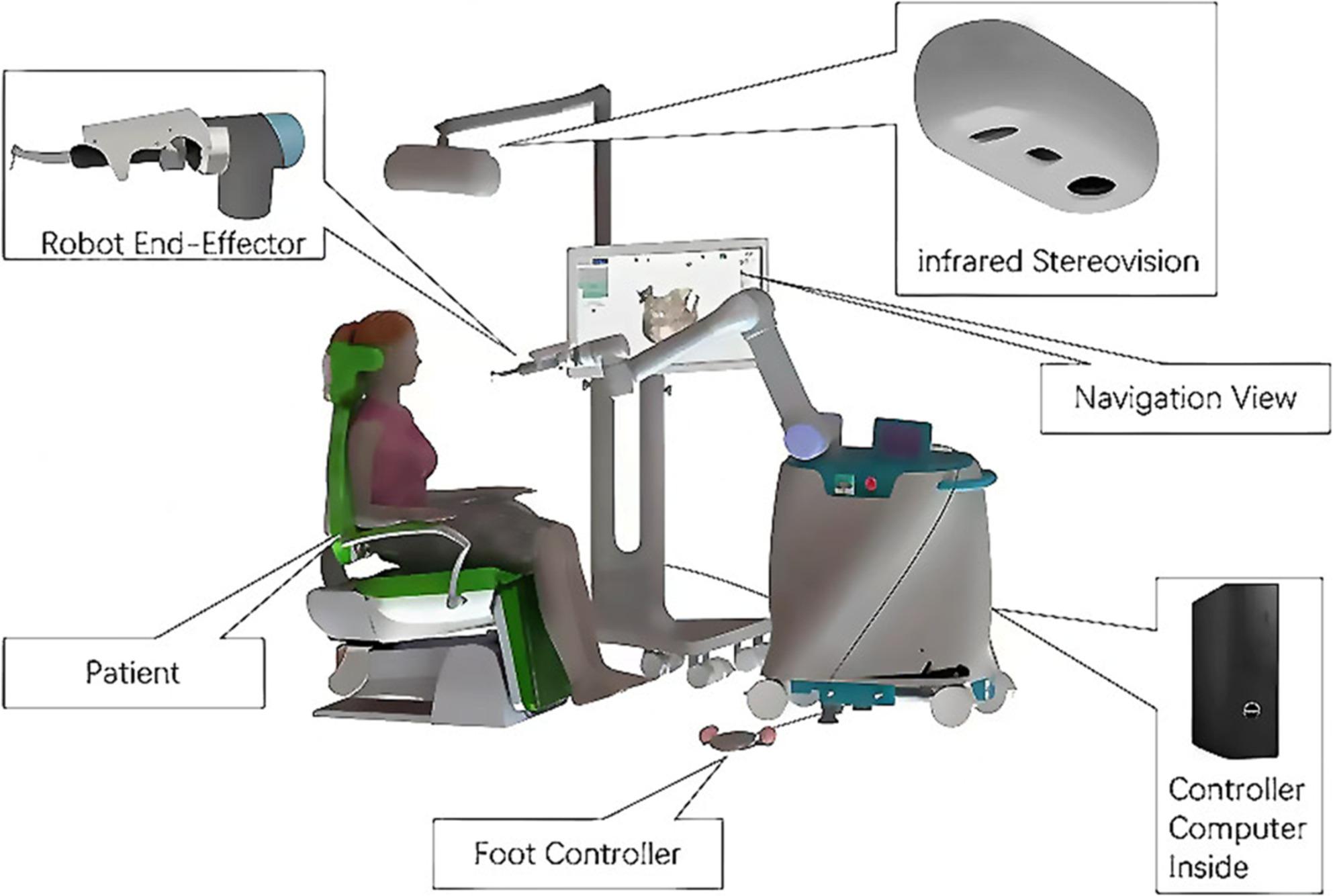
Yazhi Dental Implant Robotic System

The Yazhi Dental Implant Robotic System comprises a robot cart, a display-vision console, and a range of surgical accessories (Fig. 1). Its control unit processes all surgical data, issues motion commands, and directs the robot arm’s movements with real-time feedback, minimizing tremors and ensuring precise, stable implant placement.

The system employs optical tracking for positioning and calibration. Its FusionTrack 250 optical tracking module (Atracsys LLC, Switzerland), which comprises two cameras, simultaneously observes reflective and active fiducials (IR-LEDs) and calculates their positions via triangulation, achieving a non-interpolated measurement rate of 120 Hz. When multiple fiducials are fixed to a marker, the system accurately determines its spatial pose (position and orientation) with six degrees of freedom (x, y, z, α, β, γ).

### Surgical protocols

#### Guide group

Patients received comprehensive preoperative care, including full-mouth scaling and periodontal treatment as needed. CBCT scans (Sirona 3D, Germany) and intraoral scans (Carestream CS3700, Shanghai, China) were performed, with data imported into DENTAL CS3700 software for registration. The surgical guides were designed based on functional and restoration-oriented principles and printed by a NextDent 5100 3D printer (3D SYSTEMS, Rock Hill, SC, USA).

The 3D-printed guide was properly placed on residual teeth (finger pressure fixation for the guide group; adhesive bonding for the robot group) after disinfection. Osteotomy preparations were performed by a senior implantologist under anesthesia, followed by Osstem TSⅢ SA (OSSTEM Co., South Korea) implant placement and healing abutment connection. Postoperative CBCT scans were analyzed using Dentiq Guide (3D Industrial ImagingCo., Ltd. South Korea) software to measure deviations, which were then documented. Restorations were completed three months after surgery.

#### Robotic group

**Fig. 2:**
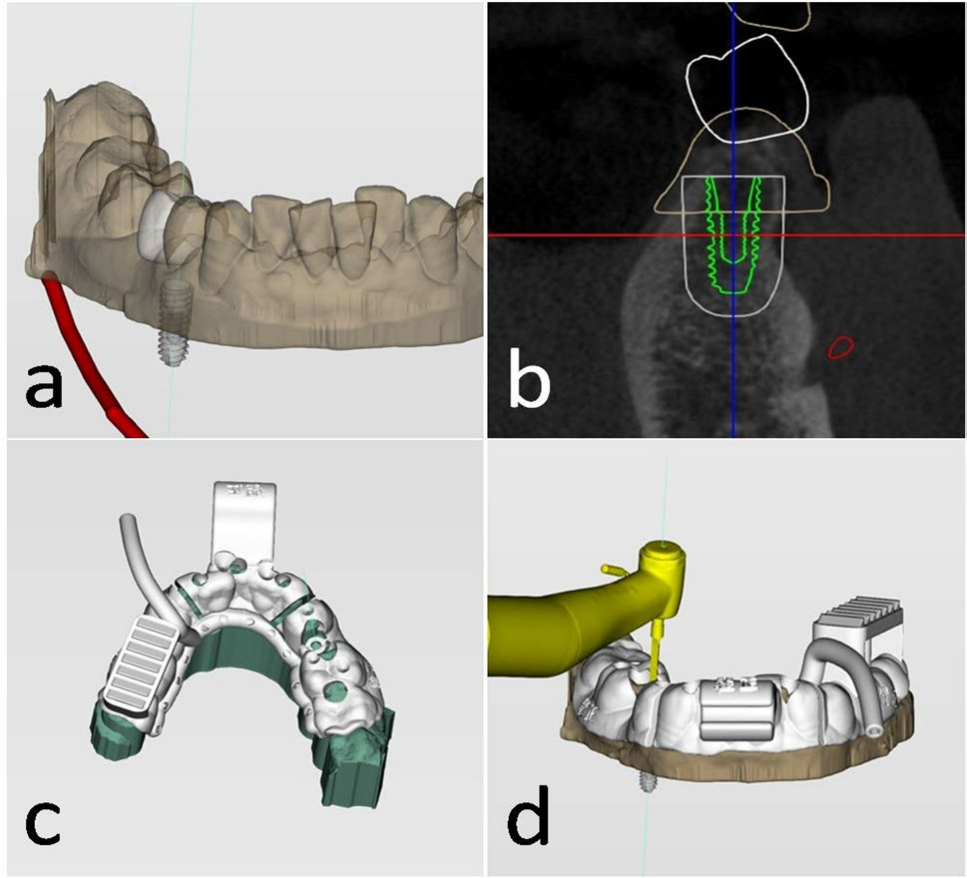
Robotic planting surgery planning. **a** Restoration plan, **b** Implantation plan, **c** Guide designing, **d** Guide registration

Preoperative CBCT and intraoral scanning (Carestream DENTAL CS3700) were conducted similarly. Data were imported into DentalNavi software (Yake Wisdom, Beijing, China) for registration and landmark identification, including the mandibular canal and maxillary sinus. Custom surgical guides and mouth openers were designed, printed, and used with registration fixtures and marker connectors (Fig. 2).

Calibration procedures were performed by the following steps:

1. Upon importing the CT and scanning data, select at least three widely spaced fiducial points on the scanned images to achieve precise registration.
2. Select the appropriate implant type and plan the implant position, then verify the surgical protocol with the software.
3. Manually designate at least three registration points on stable natural teeth. These points should be evenly distributed across the dental arch.
4. Initiate registration to complete the calibration process.

**Fig. 3:**
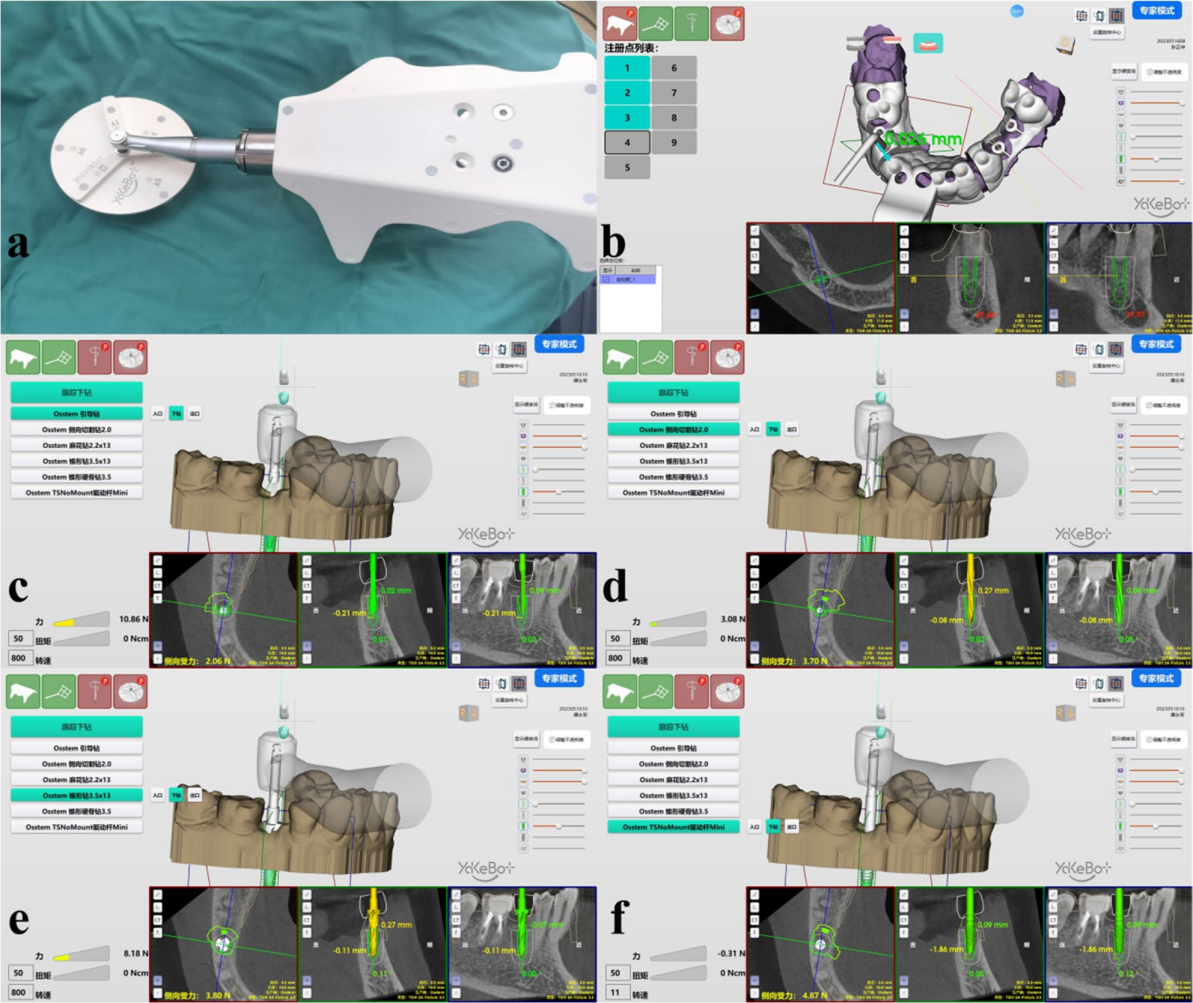
Robotic planting surgery process. **a** Probe calibration, **b** Position check, **c** Intraoral positioning, **d**-**e** Stepwise osteotomy, **f** implant placement

The surgeon planned personalized osteotomy sequences based on patients’ bone density and morphology (Table 3.). Under local anesthesia, a positioning fixture with a marker was bonded intraorally. Probe accuracy checks and handpiece trajectory registrationwere performed according to manufacturer instructions. During surgery, the robotic handpiece performed osteotomies under real-time 3D navigation, with the surgeon controlling drilling depth and angle via foot pedals (Fig. 3). After implant placement, healing abutments were manually installed. (Table 4)

**Table 3:** An example of personalized surgical plan

Seq.	Drill Type	Drill Model	Speed(rpm)
1	Osstem Lance drill	AGDLC	1200
2	Yake Depth Measurment drill	YKB-DD20026	800
3	Osstem Twist drill	2.2*11.5	800
4	Osstem Sidecut drill	3.0	800
5	Osstem Taper drill	3.5*11.5	800
6	Osstem Taper drill	4.0*11.5	800
7	Osstem Cortical bone drill	4.0	800
8	Osstem Implant Drive	— —	50

**Table 4:** Comparison of angle deviation

	Angle Deviation/Degree		
	Bucco-lingual	Mesio-distal	Global deviation
Guide group (*n* = 31)	1.289 ± 1.067	1.652 ± 1.421	2.315 ± 1.464
Robot group (*n* = 32)	0.483 ± 0.334	0.509 ± 0.426	0.775 ± 0.404
t	4.108	4.281	5.638
P	< 0.01	< 0.01	< 0.01

**Fig. 4:**
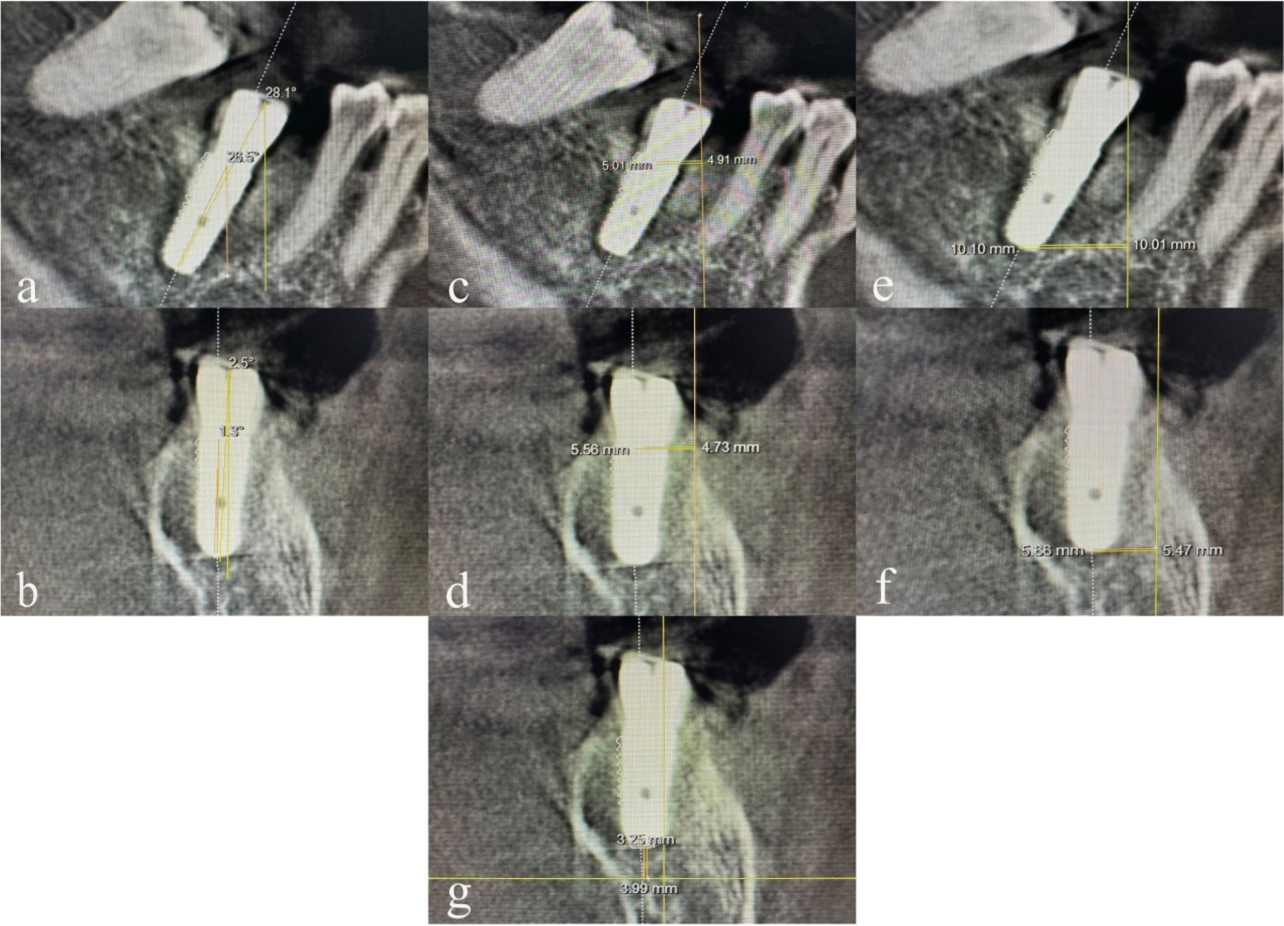
Postoperative evaluation of deviations in two directions. Planned positions were shown as white contour **a** Mesio-distal angular deviation, **b** Bucco-lingual angular deviation, **c** Mesio-distal cervical distance deviation, **d** Bucco-lingual cervical distance deviation, **e** Mesio-distal apical distance deviation, **f** Bucco-lingual apical distance deviation, **g** Depth discrepancy

Postoperative CBCT data were analyzed against the planned positions for deviations (Fig. 4).

### Data collection and analysis

Comparing the postoperative implant position with the preoperative virtual plan involves the following three steps. First, the preoperative CT data (DICOM format), the planned 3D implant model (STL format), and the postoperative CT data are imported into the DentalNavi oral surgical planning software. Second, a reference basis is selected. The key to software-based registration is identifying stable anatomical landmarks or fiducial markers that remain unchanged between the pre- and postoperative datasets. Examples include bony landmarks (the mandibular canal, mental foramen), specific features of adjacent teeth (as selected in this study), or custom-fabricated positioning guides. The algorithm then performs a best-fit alignment to superimpose the preoperative and postoperative 3D datasets based on selected reference points. Third, once the reference points are successfully aligned, indicating registration completion, the positions (or axes) of the implants can be projected and measured.

Cervical, apical and angular deviations between planned and actual implant position were projected into two directions, bucco-lingual and mesio-distal (Fig. 4). The bucco-lingual reference plane is defined as the plane that exactly passes through the center of the implant central screw in the bucco-lingual direction, so as the mesio-distal reference plane.

All over, seven parameters were employed to reflect the difference between the two groups: bucco-lingual and mesio-distal angle deviation, bucco-lingual and mesio-distal distance at cervical level, bucco-lingual and mesio-distal distance at apical level and depth discrepancy. Statistical analysis was conduct on each parameter between two groups.

Data of the groups were measured three times by the same operator to ensure accuracy. Both groups’ postoperative scans were evaluated with DentalNavi software, focusing on quantifying deviations in various positions.

### Statistics

All data were analyzed using SPSS 17.0. Intergroup comparisons employed t-tests, with variance homogeneity checked via chi-square tests; for multiple small-sample groups, ANOVA was used where appropriate. Significance was set at *P* < 0.05, with *P* < 0.01 indicating highly important results.

## Results

Surgical procedures were successfully completed in both the robot-assisted and static guide-assisted groups, with postoperative CBCT data fully acquired for all 60 enrolled patients.

The analysis showed that robotic placement considerably outperformed guide-supported procedures in terms of implant angle in both bucco-lingual and mesio-distal directions (*P* < 0.01), showing superior accuracy (Table 4). The robot group also demonstrated lower deviations in cervical and apical level in both directions (Tables 5 and 6), while depth showed no important difference (*P* > 0.05, Table 7). Importantly, robotic accuracy remained consistent across various surgical scenarios, including conventional, immediate, bone defect, class IV bone, sinus floor elevation, and multi-teeth implantations (Table 8).

**Table 5:** Comparison of cervical distance deviation

	Cervical Distance/mm		
	Bucco-lingual	Mesio-distal	Global deviation
Guide group (*n* = 31)	0.461 ± 0.310	0.441 ± 0.231	0.798 ± 0.291
Robot group (*n* = 32)	0.183 ± 0.190	0.121 ± 0.125	0.516 ± 0.274
t	4.248	6.757	3.894
P	< 0.01	< 0.01	< 0.01

**Table 6:** Comparison of apical distance deviation

Apical Distance/mm			
	Bucco-lingual	Mesio-distal	Global deviation
Guide group (*n*=31)	0.508 ± 0.330	0.476 ± 0.346	0.866 ± 0.362
Robot group (*n*=32)	0.164 ± 0.154	0.135 ± 0.124	0.513 ± 0.254
t	5.238	5.147	4.417
*P*	<0.01	<0.01	<0.01

**Table 7:** Comparison of depth discrepancy

	Guide group (*n*=31)	Robot group (*n*=32)	t	*P*
Implant depth error	0.426 ± 0.205	0.335 ± 0.029	1.339	0.19

**Table 8:** Comparison of the three-dimensional implant position in different surgical scenarios

Robot Group			
	Angle error/degree	Cervical distance/mm	Apical distance/mm
Conventional imp(*n*=6)	0.914 ± 0.558	0.652 ± 0.302	0.678 ± 0.290
Immediate imp(*n*=9)	0.821 ± 0.414	0.468 ± 0.223	0.456 ± 0.216
Bone defect(*n*=4)	0.778 ± 0.283	0.581 ± 0.359	0.614 ± 0.318
Type IV bone (*n*=5)	0.813 ± 0.308	0.600 ± 0.310	0.573 ± 0.215
maxillary sinus lift (*n*=4)	0.462 ± 0.308	0.465 ± 0.080	0.452 ± 0.074
multi-teeth implantation (*n*=4)	0.878 ± 0.239	0.260 ± 0.080	0.275 ± 0.021
Variance Homogeneity（χ2）	0.899	2.877	5.442
F	0.609	1.151	1.693
*P*	0.69	0.36	0.17

## Discussion

The dental implant robotic system utilized in this study successfully completed the placement of 32 implants in 30 patients across varied clinical scenarios, confirming its clinical applicability in diverse surgical environments. According deviation analyze, robotic system significantly improves the angle and distance deviation, especially in mesio-distal direction which decreases over 70% among angle, cervical and apical comparison with guide system. Meanwhile, comparison of bucco-lingual direction witnesses a decrease of over 60% in deviation across various surgical scenarios. These improvements are mostly attributed to precise real-time digital calibration and intraoperative feedback adjustment of robotic system. Our data does not show remarkable difference in depth deviation between the two groups. This result probably stems from the stop ring structure used in both groups.

Robot-assisted implantation built upon the foundation of static guides, therefore inheriting its accuracy advantages. In this study, the mean angular deviation using the robotic system was 0.775 ± 0.404°, while the mean deviation at the cervical, apical levels and depth deviation was 0.798 ± 0.291 mm, 0.886 ± 0.362 mm and 0.335 ± 0.029 mm respectively. These results shows light variations compared to some previous reports [19, 20].Inanin vitro simulation study, Xia et al. reported that robotic system achieved a mean angular deviation of 1.03 ± 0.56°, with platform and apex deviations of 0.69 ± 0.29 mm and 0.74 ± 0.27 mm respectively [19]. This difference may result from the uniformity of in vitro models, which reduced complexity of distance control. Wang et al. in a clinical study on edentulous patients, reported mean cervical, apical, depth and angular deviations of 0.65 ± 0.25 mm, 0.65 ± 0.22 mm,0.49 ± 0.24 mm and 1.43 ± 1.18° for robot-assisted implantation [20]. The reduced distance deviation could be attributed to its exclusive focus on edentulous jaws implantation, representing a more uniform surgical scenario. Meanwhile, the author also reported a robot superiority over static guide in deviation control.

Unlike previous studies [21, 22], angle and distance deviations in this study are divided in two directions, bucco-lingual and mesio-distal. In this way, we are able to reflect the deviation with different highlights according to various scenarios. The posterior teeth implantation, which suffered most from mesio-distal and depth deviation under freehand surgery [23, 24], pays more attention in these directions for defects of mesio-distal position may result in bad occlusion curve and mistakes of depth control can bring severe damage to mandibular nerve [25]. In contrast, anterior teeth implantation gives its priority to angular and bucco-lingual deviations [26], in which case an error in angle performance usually leads to aesthetic and function failure.

Static guidance and robot implantation system basically overcome these freehand defects by fixing the drills with guide or robot arm. It is reported [27]that static guide achieves a linear accuracy within 1 mm and angular accuracy ranging from 2°to 3°.

Jia et al. achieved in a clinical study a cervical and apical deviation of 1.31 ± 0.62 mm and 1.47 ± 0.65 mm, which were higher than in this study [28]. In another research on edentulous implantation, Chen et al. reported 0.97 ± 0.64 mm and 1.40 ± 0.82 mm for cervical and apical deviation, and 3.44 ± 2.78°for angular deviation [29]. This variation demonstrated that static guide effect was vulnerable to several factors such as guide manufacture and implantation scenario.

The robotic system takes a step forward via real-time intraoperative adjustment to obtain better error control [30], but its advantages in precise implantation remain unclear.

The comparison of these results with static navigation implantation is consistent with findings from earlier studies and meta-analyses [1, 31]. This superior performance results from the combined advantages of the robotic system. The real-time tracking capability, based on optical calibration and CBCT data model fitting, enables the robotic arm to comply with patient posture continuously and maintain a stable position at any angle, which ensured the robot a better repeatable accuracy [1, 32] and maximized the utilization of available bone volume [1, 2]. These capabilities brought greater flexibility and adaptability to diverse surgical conditions and patient anatomies for the robotic system [31]. Previous research has indicated that static guide-based navigation surgery shows no significant differences in accuracy between the maxilla and mandible, flap and flapless surgery, or anterior and posterior region implantations [33, 34]. Therefore, this study covered various types of implantation cases to thoroughly assess the robotic system performance in realistic clinical contexts.

It should be noted that robot-navigated implantation, as an emerging technology, is built upon the successful application of static guides [35, 36]. The accurate robotic arm operation relies on the correct position of dedicated intraoral guide. Furthermore, intraoral calibration, robotic arm path learning, and managing unexpected situations arising from patient anxiety impose higher demands on surgeons. In this study, the clinical application of the robot takes a senior surgeon over 4 weeks of pre-practice and simulation on models, posing a greater challenge for junior surgeons. Due to the study timeframe, this research could not afford to compare postoperative complications or long-term patient satisfaction between robotic and conventional implantation; these conclusions require longer-term studies.

It is foreseeable that the advancement of artificial intelligence will integrate with robot-assisted implantation to conceive optimal treatment plans [37].By accurately mapping patient anatomy and providing real-time feedback, AI will reduce reliance on clinical experience, enhance implant stability, and mitigate malposition risks, thereby further minimizing human error [38].Furthermore, trained and enhanced AI systems will autonomously perform time-consuming tasks such as implant planning and path design. By guiding clinicians along predefined pathways, they shorten surgical duration, reduce manual adjustments, and ultimately improve surgical efficiency [39].

Although initial implementation of AI technology requires substantial infrastructure and training investment—potentially incurring high costs—its long-term benefits are significant. Improved diagnostic accuracy and surgical precision through AI can reduce complications such as implant failure and infection. As AI becomes more widespread, economies of scale will lower costs, making advanced implant procedures more accessible [39, 40].

## Limitations

We acknowledge several inherent limitations in this study, which was among the first clinical applications of the robotic implant system by our team.

First, the patient cohort was limited in size and recruited exclusively from a single institution. As a newly introduced advanced medical device, public acceptance of robotic system remains low, and its use is restricted to well-equipped hospitals due to stringent operational requirements. These factors limit the generalizability of our findings to broader populations.

Second, due to the limited sample size and study duration, we were unable to perform separate error analyses for specific surgical scenarios—such as bone defect or sinus lift.

Third, to ensure procedural consistency, all surgical procedures were performed by a single experienced surgeon. Nevertheless, the learning curve associated with adopting the robotic system may have introduced performance bias.

Finally, although the deviation analysis of the robotic system met our expectations, feedback for optimizing its programming and algorithms—such as long-term patient follow-up, prosthetic outcomes, and rates of mechanical or biological complications—requires further long-term evaluation. Our team is currently planning extended studies on robotic-assisted implantation. We believe these limitations will be progressively addressed as robotic implant systems become more widely adopted and accessible.

## Conclusion

In summary, this study demonstrates that the robot-assisted implant system significantly enhances placement accuracy compared to static guide-assisted surgery, particularly in angular and linear deviations at the cervical and apical levels in both bucco-lingual and mesio-distal directions. The robotic system maintained consistent precision across a variety of surgical scenarios, confirming its clinical adaptability and reliability. This superiority largely attributable to its real-time tracking, dynamic feedback, and stable execution. However, no significant difference was observed in depth control, likely due to the mechanical constraints shared by both systems. Future studies should focus on long-term clinical outcomes, including postoperative complications and patient satisfaction.

## Data Availability

The data that support the findings of this study are available from the corresponding author upon reasonable request.

## Abbreviations

CBCT: Cone Beam Computed Tomography
3D: Three Dimensions
BL: Bucco-Lingual
MD: Mesio-Distal
GBR: Guided Bone Regeneration
ANOVA: Analysis of Variance
